# Acknowledgment to Reviewers of *Animals* in 2021

**DOI:** 10.3390/ani12030341

**Published:** 2022-01-30

**Authors:** Animals Editorial Office

**Affiliations:** MDPI AG, St. Alban-Anlage 66, 4052 Basel, Switzerland

Rigorous peer-reviews are the basis of high-quality academic publishing. Thanks to the great efforts of our reviewers, *Animals* was able to maintain its standards for the high quality of its published papers. Thanks to the contribution of our reviewers, in 2021, the median time to first decision was 17 days, and the median time to publication was 40 days. The editors would like to extend their gratitude and recognition to the following reviewers for their precious time and dedication, regardless of whether the papers they reviewed were finally published:
Abade Dos Santos, Fábio AlexandreLewczuk, DorotaAbbate, Jessica MariaLewis, HelenAbd El Wahed, AhmedLewis, RichardAbd El-Wahab, AmrLey, Jacqueline M.Abdel-Glil, MostafaLeyva-Jimenez, HectorAbdollahi-Arpanahi, RostamLi, ChangxiAbecia, José AlfonsoLi, FuchangAbegglen, Lisa M.Li, GuomingAbeni, FabioLi, HuiAbia, Akebe Luther KingLi, JunyouAbidu-Figueiredo, MarceloLi, ShoujunAblondi, MichelaLi, XiangAbudabos, Alaeldein M.Li, XiaoyuAbugomaa, AmiraLi, XinhaiAcharya, Kamal RajLi, XinweiAcharya, MohanLiang, AixinAchino, Katia FrancescaLiao, PengAcin, CristinaLiao, XindiAcutis, Pier LuigiLicata, PatriziaAdamczyk, KrzysztofLichtenwalner, AnneAdamiak, ZbigniewLicitra, RosarioAdamski, MarekLiedtke, Katarzyna G.Addie, DianeLiermann, WendyAdedokun, TayoLiesegang, AnnetteAdewole, DeborahLill, AlanAdhitya, YudhiLim, Han KyuAfonso, FernandoLima, Fábio S.Aftab, UsamaLin, Chao-NanAgnew, Dalen W.Lin, Chen-SiÁguila, LuisLin, Chun-MingAguirre, AlonsoLin, HaiAhern, Benjamin J.Lin, Hui-ChuAhlstrøm, ØysteinLin, Jin-SengAhmed, IbrahimLin, TaoAiken, GlenLin, YangAkins, MattLin, Yu-HungAkiyama, ToyokoLindholm, AmandaAkter, ShirinLindinger, MichaelAlarcon-Rojo, Alma D.Lines, Jeffrey A.Albarella, SaraLinhart, OtomarAlbayrak, HarunLinhoss, John E.Albertini, MariangelaLinster, MartinAlbrecht, ElkeLiordos, VasiliosAlbright, Julia D.Liotta, LuigiAlcalde, Maria J.Lipiński, KrzysztofAlders, Robyn G.Lipták, NándorAlegre, BeatrixLis, MarcinAlegria-Moran, RaulLisiecka, UrszulaAleri, JoshuaLittin, KateAlexander, Brenda M.Liu, Cheuk LunAlexandre, FellousLiu, EnqiAlfaro-Córdoba, MarcelaLiu, FanAlfaro-Núñez, AlonzoLiu, JundiAlfonso, SébastienLiu, QiangAli, AhmedLiu, RunxiaAli, Ali A.Liu, ShiminAlibhai, Hatim I. K.Liu, WeiweiAlić, AmerLiu, YunAlizadeh, MohammadaliLiu, ZhaoyingAlkhtib, AshrafLiuti, TizianaAllard, StephanieLjubojevic, DraganaAllegretti, Silmara MarquesLlavanera, MarcAllen, DanielLlonch, PolAllepuz Palau, Alberto OscarLloyd, HuwAlleva, EnricoLloyd, JaniceAllworth, BruceLoberg, JennyAl-Marzooqi, WaleedLobo-da-Cunha, AlexandreAlmeida, ArlindoŁochyńska, MałgorzataAlmiron, NúriaLockwood, RandallAloisi, Anna MariaLoh, AndrewAlonge, SalvatoreLoiseau, NicolasAlonso, Marta E.Lokhorst, KeesAlonso, Marta LópezLombardero, MatildeAlvarenga, Marco AntônioLombardi, JasonAlvarez, JavierLombardi, PieroAlvarez-González, Carlos AlfonsoLombardi, PietroAlvarez-Narvaez, SonsirayLomillos Pérez, Juan ManuelÁlvarez-Rodríguez, ManuelLong, MengxianAmaral, Andreia J.Long, YongAmasheh, SalahLooper, Michael L.Amato, Monica GuarinoLopes, Júlio C.Ambrose, DivakarLopes, Luciano BastosAmbrosini, Yoko M.Lopes, Maria SusanaAmici, AndreaLopes, Ricardo JorgeAmiridis, GeorgeLópez Albors, OctavioAmirpour-Najafabadi, HamedLópez Parra, María MontañaAmores, GustavoLópez, Bruno DíazAmorim, Renee LauferLópez, CeferinoAndersen, MonicaLópez, José ManuelAnderson, Kenneth E.Lopez-Bote, Clemente J.Anderson, KevinLópez-Cepero Borrego, JavierAnderson, Nichole C.López-Cortegano, EugenioAndersson, MagnusLópez-Gatius, FernandoAndersson, RobbyLopez-Lorenzo, GonzaloAnderwald, PiaLópez-Sanmartín, MonserratAndrade-Montemayor, Héctor MarioLopez-Sanroman, JavierAndrás, GáspárdyLopreiato, VincenzoAndré, Marcos RogérioŁopucki, RafałAndreani, GiuliaLora, IsabellaAndreani, Nadia AndreaLorenz, IngridAndreella, AngelaLorenzo, Jose M.André-Fontaine, GenevieveLoreti, PierpaoloAndren, HenrikLosada, AnaAndreoni, FrancescaLososová, JanaAndreoni, ValentinaLoudon, Kate M. W.Andres, KrzysztofLourenço, MartaAndrighetto, IginoLoux, Shavahn C.Angelici, Maria CristinaLow, Wai YeeAngeoletto, Fábio Henrique SoaresLucchese, LauraAnge-van Heugten, KimberlyLuchiari, Ana CarolinaAnglani, FrancaLuciani, AlessiaAngrecka, SabinaLucini, CarlaAnneberg, IngerLucon-Xiccato, TyroneAntal, LászlóLucy, Matthew C.Antanaitis, RamunasLudlow, MartinAnthony, Brandon P.Ludwiczak, AgnieszkaAntonacci, RacheleLugar, DrewAntoniassi, RosemarLuis Pereira, GuilhermeAntonino, José DijairŁukasiewicz, MonikaAntunović, BorisLuković, ZoranAntzoulatos, EvanLundeheim, NilsAnusz, KrzysztofLunge, Vagner RicardoAparicio, Miguel A.Luo, XinApt, WernerLupo, Karen D.Aqib, Amjad IslamLuridiana, SebastianoAragão, CláudiaLürzel, StephanieAragunde-Kohl, UrsulaLuther King, Abia AkebeArango Duque, GuillermoLuther, Anne-MarieAraújo, MargaridaLüttgenau, JohannesArchibald, AlanLutz, CorrineArciszewski, MarcinLuzi, FabioArculeo, MarcoLymperopoulos, AristotelisArczewska-Włosek, AnnaLynch, EmilyArfuso, FrancescaMa, GangArgente, María-JoséMaak, SteffenArgüello, AnastasioMabjeesh, Sameer J.Arias, Rodrigo A.Maccatrozzo, LisaAriyarathna, HarshaMacedo Barragán, Rafael JulioArlt, SebastianMacedo, Gustavo GuerinoArmando, FedericoMacias Cruz, UlisesArmengol, RamónMacLeod, IonaArmengou, LaraMacNamara, MaureenArriagada, GloriaMacNicol, Jennifer L.Arriaga-Jordan, Carlos ManuelMacrì, FrancescoArsenault, JulieMadeira De Carvalho, Luis ManuelArsenopoulos, KonstantinosMadkour, AichaArsenos, GeorgiosMadsen, Johannes GulmannArthington, John D.Madsen-Bouterse, Sally A.Arukha, Ananta PrasadMaduna, SimoAsai, TetsuoMaeda, TaroAsakawa, MitsuhikoMaertens, LucAsher, GeoffMagalhães, AnaAsproni, PietroMagrin, LuisaAssmann, Tangriani SimioniMahony, Timothy JohnAtes, SerkanMahsoub, HassanAthanasiou, Labrini V.Mai, DacAttia, Youssef A.Maier, GabrieleAttili, Anna RitaMaier, KristinaAtxaerandio, RaquelMain, DavidAudano, MatteoMaiorka, AlexAuer, HerbertMajecka, KatarzynaAungier, SandraMajewska, Małgorzata P.Aurich, ChristineMakagon, Maja M.Aurich, JoergMakecha, RadhikaAvalos, ArianMakhutova, OlesiaAvenant-Oldewage, AnnemariéMakowiecki, DanielAvilés Ramírez, CarmenMakraki, Maristela C.Avondo, MarcellaMalak-Rawlikowska, AgataAwruch, CynthiaMalandrakis, EmmanouilAzab, WalidMałgorzata, ZiarnoAzain, MichaelMallory, Frank F.Azarpajouh, SamanehMalmuthuge, NilushaAzkona, GarikoitzMammi, LudovicaBaars, TonMamuris, ZissisBabicz, MarekManafiazar, GhaderBabol, JakubMañanós, EvaristoBach, LotharMancia, AnnalauraBach, LydiaMancianti, FrancescaBachmann, MartinMancin, EnricoBackus, Brittany L.Mancini, EmilianoBacon, HeatherMancuso, FrancescaBadino, PaolaManenti, RaoulBadiola, IgnacioManfredi, Maria TeresaBagnicka, EmiliaManiaci, GiuseppeBailey, Chad M.Mankin, Richard W.Bailoni, LuciaMannelli, FedericaBajcsy, ÁrpádManoharan-Basil, Sheeba S.Bajer, AnnaManriquez, DiegoBaker, Kate C.Mans, ChristophBaker, LivMansell, PeterBakker, DouweManso, TeresaBalasubramaniam, KrishnaManso-Díaz, GabrielBalasubramanian, BalamuralikrishnanManu, HayfordBalčiauskas, LinasManuelian, CarmenBalčiauskienė, LaimaMarana, Moonika HaahrBálint, AnnaMaranesi, MargheritaBalocchi, OscarMarcato, Simara MarciaBalogh, OrsolyaMarchán, Daniel F.Baluska, FrantisekMarchegiani, AndreaBalzani, AgneseMarchesini, GiorgioBanaszewska, DorotaMarcinčáková, DanaBańbura, Marcin W.Marco, DaddaBancerz-Kisiel, AgataMarco, RussoBandara, Rathnayaka Mudiyanselage Amila SubhashinieMarek, LukaszewiczBani, PaoloMargerison, JeanBaotic, AntonMargetín, MilanBaragli, PaoloMargulis, SusanBarański, WojciechMaribo, HanneBarba, EmilioMarín Carrillo, PedroBarbagianni, MarianaMarín Orenga, ClaraBarbas, João PedroMarin, Clara M.Barbera, SalvatoreMarín-García, Pablo JesúsBarberio, AntonioMarini, DanilaBarbu Tudoran, LucianMarino, GabrieleBarcena, CarmenMarinović, ZoranBard, Kim A.Markovic, KristinaBardi, EdoardoMarliani, GiovannaBarja, Juan L.Marlow, ClaytonBarnard, ShanisMármol-Sánchez, EmilioBaro, Jesus A.Marongiu, Maria LauraBarone, Carmela M. A.Marques Costa Flaiban, Karina KellerBarrachina, LauraMarques, Maria Do Rosário FernandesBarragan, Adrian A.Marques, MarianaBarrasso, RobertaMarquez-Lara, Alejandro J.Barreiros, João PedroMarquis, VirginieBarreras-Serrano, AlbertoMarreros, NelsonBarrionuevo Jimenez, Francisco JavierMarshall, KarenBarros-Velázquez, JorgeMarshall, LindsayBarry, GeraldMarsilio, FulvioBarszcz, MarcinMarszałek, MartaBarta, EndreMartín Castillo, MargaritaBartkovský, MartinMartin, AdamBartlow, Andrew W.Martin, JessicaBartolomé, JordiMartin, KarinBartolommei, PaolaMartín, Natalia P.Barton, Ann-KristinMartin-Campos, Jesus M.Barton, DiMartín-Cuervo, MaríaBasaglia, FedericaMartinez, DiegoBasak, AninditaMartínez, José MarioBasheer, FaizaMartínez, Tomás FranciscoBasini, GiuseppinaMartínez, YordanBasioura, AthinaMartínez-López, VicenteBąska, PiotrMartínez-Pérez, PedroBastos, ArmandaMartínez-Porchas, MarcelBates, JohnMartino, GiuseppeBathgate, RoslynMartino, Piera AnnaBatista, SóniaMartins, Ana D.Batkowska, JustynaMartins, AngelaBatorska, MartynaMartins-Bessa, AnaBattaglini, Luca MariaMartos Martinez-Caja, AnaBatts, William N.Martuzzi, FrancescaBauman, Cathy A.Mas, AliciaBaumgartner, KatrinMascaro, MaiteBaur, MarkusMas-Coma, SantiagoBawm, SawMaselli, ValeriaBaxter, Greg S.Mashkour, NargesBayfield, Oliver W.Masino, FrancescaBaynes, RonaldMasiulis, MariusBażanów, BarbaraMaślanka, TomaszBeasley, ErinMassimini, MarcellaBeaver, BonnieMastellar, Sara L.Bebbere, DanielaMateo, JavierBeck, MatthewMateos, IvánBecker, DoreenMatera, Julia MariaBecker, JensMateus, Luísa MariaBeddoe, TravisMateus, TeresaBedore, Jessica SuageeMathurkar, ShashwatiBeer, SvenMatics, ZsoltBeever, JonMatikonda, SiddharthBeggs, David S.Mátis, GáborBeineke, AndreasMatsubara, HajimeBeitz, Donald C.Matsuda, FukoBelli, Carla BargiMatsuhisa, FumikazuBelli, ManuelMatsuwaki, TakashiBellini, LucaMatthews, LindsayBellini, SilviaMattiello, SilvanaBello, Nicholas T.Maunsell, FionaBelotindos, LawrenceMaurer, PatricBeltman, MarijkeMaurício, Ana ColetteBeltrán-Frutos, EsterMaurin, MaxBełżecki, GrzegorzMavrogianni, Vasia S.Benazzi, CinziaMavrommatis, AlexandrosBenchaar, ChaoukiMawson, Peter R.Bendaoud, MeriemMaxwell, HerrisBenedetti, YaninaMaxwell, Thomas M. R.Benissa, SaidMayhew, Jessica A.Benítez, RitaMazgajski, Tomasz D.Benjamin, MadonnaMazurek-Popczyk, JustynaBennato, FrancescaMazurkiewicz, JanBennett, PauleenMazur-Kuśnirek, MagdalenaBennitt, EmilyMazzola, Silvia MichelaBentosela, MarianaMazzone, PieraBenyhe, SandorMazzotta, ElisaBenzertiha, AbdelbassetMbajiorgu, Christian A.Bergadano, AlessandraMcBride, AnneBergen, WernerMcCarthy, Timothy C.Berger, AnneMcClure, MatthewBergh, ØivindMcCoski, SarahBerglund, AlixMcCrackin, Mary AnnBergmann, ShanaMcDaneld, Tara G.Bergvall, Kerstin E.McDermott, KatieBerliner, ElizabethMcDonnell, Sue M.Bermejo-Poza, RubénMcFarlane, Zachary D.Bernhardt, HeinzMcGee, MarkBernier Gosselin, VéroniqueMcGuire, BettyBernstein, MarkMcLellan, Micheal A.Bert, WimMcManus, PhilBertechini, Antônio GilbertoMcMichael, Maureen A.Bertelloni, FabrizioMcMillan, FrankBerteselli, Greta VeronicaMcPherson, Andrew S.Bertolín Pardos, Juan RamónMead, RebeccaBertoni, Giuseppe (Italy)Medeiros Da Silva, GleiseBertoni, Giuseppe (Switzerland)Medger, KatarinaBertrand, Deputte L.Medica, PietroBęś, AgnieszkaMedina-Reyes, Estefany IngridBester, LindaMedrano, Juan HernandezBeugnet, FrédéricMeier, JacquelineBevan, ElizabethMejdell, CecilieBhatti, Muhammad AzherMejía-Ruíz, Claudio HumbertoBibbal, DelphineMekuchi, MiyukiBibbiani, CarloMeléndez Suárez, DanielaBibbo, JessicaMelgarejo, TonatiuhBicudo, Álvaro J. A.Melzer, NinaBielański, PawełMenanteau-Ledouble, SimonBielas, WiesławMenardo, SimonaBiermann, Nora M.Menchetti, LauraBiesek, JakubMendes, ThiagoBilal, GhulamMendoza García, Francisco JavierBillenkamp, FabianMeneguz, MarcoBillinis, CharalambosMensah, Enoch A.Binder, AprilMenten, José Fernando MachadoBiscarini, FilippoMercader, JosepBisi, FrancescoMercati, FrancescaBittar, Carla Maris MachadoMerella, PaoloBlache, DominiqueMerino, Eva Maria PerezBlackman, Simone A.Merino, SantiagoBlaha, ThomasMerkies, KatrinaBland, IanMerlo, BarbaraBlank, RalfMeroni, GabrielleBlatchford, Richard A.Mesquita, JoãoBleach, EmmaMessina, SimoneBlikslager, AnthonyMethion, SeverineBlock, HushtonMetodiev, NikolaBlock, JannaMetrione, LaraBloom, Peter H.Meurer, FábioBloor, Juliette M. G.Mezzetti, MatteoBobbo, TaniaMiazek, ArkadiuszBockstahler, BarbaraMicek, PiotrBoelhauve, MarcMichaels, ChristopherBoere, VannerMichalczuk, MonikaBoerman, Jacquelyn P.Michie, CraigBogusławska-Tryk, MonikaMichl, Stéphanie CélineBoiani, MicheleMiciński, JanBókony, VeronikaMiddelkoop, AnouschkaBolcato, MatteoMielcarek-Bocheńska, PaulinaBoles, Jane AnnMies, MiguelBollen, PeterMigdał, ŁukaszBombik, ElżbietaMiglino, Maria AngelicaBonacic, Cristián F.Miklosi, AdamBonadonna, AlessandroMikołajczak, BeataBonato, MaudMikuła, RobertBonfante, FrancescoMikulski, DariuszBongalhardo, Denise CalistoMikusinski, GrzegorzBongiovanni, LauraMilani, ChiaraBoni, RaffaeleMillen, Danilo DominguesBonilla González, EdmundoMiller, David S.Bonos, EleftheriosMiller, IngridBooth, DavidMiller, Jeffrey D.Boqvist, SofiaMiller, MicheleBordehore, CesarMiller, TrishBordin, LucianaMills, ErezBorg, BertilMills, PaulBork, Edward W.Miniero, RobertoBorowski, ZbigniewMiniscalco, BarbaraBortolami, AlessioMinnie, LiaanBosch, JaimeMinozzi, GiuliettaBoschiroli, María LauraMinucci, SergioBoselli, CarloMiocinovic, JelenaBosi, PaoloMioduchowska, MonikaBoss, Darrin L.Miraglia, DinoBossus, Maryline C.Miranda, LuisaBotelho, AnaMiranda, MartaBotello-Álvarez, EnriqueMiranda-de La Lama, Genaro C.Botes, MarelizeMiranda-Filho, Kleber CamposBouchami, OnsMiras Mermudes, José RicardoBouchnick, RamMiretti, SilviaBoulton, KayMiró, FranciscoBourassa, Dianna V.Mironov, Sergei V.Bourette, Roland P.Mishra, BirendraBowles, DavidMisztal, TomaszBowman, Larry L.Mitchell, Mark A.Boxshall, GeoffMitchell, RobertBoyd, JacquelineMiura, RyotaroBracarense, AnaMiyakawa, HitoshiBradbery, AmandaMiyano, MasaruBradshaw, DonMiyashita, TadashiBrady, KristenMiyazawa, KohtaroBragaglio, AndreaMizunoya, WataruBrambilla, Paola G.Mizusawa, KantaBran, José A.Mladineo, IvonaBranco, PauloMłodawska, WiesławaBrandt, AdamMnisi, Caven MguvaneBrandt, Luise ØrstedMo, DelinBrandt, SabineMoallem, UziBrauchle, MichaelMoberly, Heather K.Bravo-Rivera, ChristianModesto, PaolaBray, Heather J.Mogielnicka-Brzozowska, MarzenaBrech, Susana LeMogut, DamirBreitbart, HaimMohamed, RedaBremm, CarolinaMoisan, Marie-PierreBrereton, JamesMolento, Carla Forte MaiolinoBressan, Maria CristinaMolik, EdytaBreton, Timothy S.Molina, AntonioBreur, Gert J.Møller, Steen HenrikBriceño, CristóbalMollo Neto, MarioBriefer Freymond, SabrinaMollo, AntonioBriganti, AngelaMolotsi, AnnelinBright, TerriMongillo, PaoloBrignolo, LaurieMoniello, GiuseppeBrisse, MorganMonika, Fra̧czekBrito, Nuno V.Moniuszko, HannaBritti, DomenicoMonk, Jessica E.Brodacki, AntoniMonllor Guerra, PaulaBrooman, SimonMonsees, ThomasBrotons, Jose M.Monteiro-Neto, ValérioBroucek, JanMonti, GustavoBrown, CulumMontoro-Dasí, LauraBrowning, HeatherMontoya-Alonso, J. AlbertoBrubaker, LaurenMoody, CarlyBrufau, JoaquimMoraes, CarolineBrugger, DanielMoraïs, SarahBrugiapaglia, AlbertoMorales Doreste, ManuelBrundo, Maria ViolettaMora-Medina, PatriciaBruneau, LauraMorán, PalomaBrunetti, BarbaraMorbidini, LucianoBruno, BarbaraMordenti, AttilioBrust, VeraMoreira, OlgaBrys, JoannaMorelli, GiadaBrzozowski, MarianMoreno, Ricardo D.Büchler, RalphMoreno-Millan, MiguelBuchner, FlorianMoresco, AnnekeBuckland, EmmaMoretti, RiccardoBuckley, LouiseMorfin, NuriaBuckley, PetraMorgan, KathleenBudik, SvenMorgaz, JuánBueno Dalto, DanyelMori, Claudia Madalena CabreraBueso-Ródenas, JoelMori, EmilianoBugg, WillMorkune, RasaBugno-Poniewierska, MonikaMoroni, BarbaraBugoni, LeandroMorrell, Jane M.Buhr, JeffMorrice-West, Ashleigh V.Buitenhuis, Albert JohannesMorris, Kevin N.Bungartz, GerdMorris, TimBungo, TakashiMorrison, AnnBuono, FrancescoMorrison, PhilippaBurda, HynekMorrison, SarahBurdick Sanchez, NicoleMorton, DavidBurger, PamelaMoscati, LiviaBurke, Joan M.Moscovice, Liza RoseBurkowska-But, AleksandraMossialos, DimitrisBurley, Heather KristinMotamed, CyrusBurnett, StephenMott, AlexanderBurnett, Tracy A.Mouloodi, SaeedBurrow, Heather M.Mounien, LourdesBury, StanisławMoura, ArlindoBush, Stephen J.Mouritsen, HenrikBussalleu, EvaMoustakas-Verho, Jacqueline E.Bustamante, Hedie A.Moustsen, Vivi AarestrupBustos-Martínez, JaimeMozdziak, PaulButler, DeborahMrkun, JankoButler, FidelmaMrkvicova, EvaButler, GillianMucha, MarionButts, IanMueller, Megan KielyButty, AdrienMukiibi, RobertByrd, James AllenMulas, AntonelloByrne, AndrewMullan, Siobhan M.Bystroń, JarosławMuller, AnandaCaballero, Maria JoséMüller, AnjaCaballero, Raúl PérezMullineaux, ElisabethCaballero-Villalobos, JavierMulvenna, ChristinaCabaret, JacquesMuñana, Karen RCabral De Mello, Diogo CavalcantiMunekata, Paulo E. S.Cabrera-Guzmán, ElisaMuñoz, AnaCadiergues, Marie ChristineMunoz, MariaCadogan, David J.Muñoz-Poblete, CarlosCaffara, MonicaMunsterhjelm, CamillaCagnardi, PetraMura, Maria ConsueloCakebread, PeterMurakami, HitoshiCaldas-Cueva, Juan P.Murakami, MamiCallegher, Claudio ZandonellaMurashita, KojiCalsamiglia, Sergio BlancafortMurawska, DariaCalvo, Jorge H.Murison, PamelaCamacho Chab, Juan CarlosMurr, MagdalenaCambier, CaroleMuscatello, GaryCambra Bort, Josep M.Muscatello, Luisa VeraCameron, LornaMusk, Gabrielle C.Cammilleri, GaetanoMuszyński, SiemowitCampanella, JamesMutu, Margaret ShirleyCampbell, Angus J. D.Muyyarikkandy, Muhammed ShafeekhCampbell, DanaMuzaffar, SabirCampbell, LachlanMykkänen, Anna KristinaCampera, MarcoMysterud, AtleCampomizzi, AndrewNääs, IrenilzaCanadas, AnaNääs, Irenilza De AlencarCandellone, AlessiaNagamine, YoshitakaCañon, JavierNagashima, Jennifer B.Cantarelli, ViniciusNaidu Chitrala, KumaraswamyCantón, Germán JoséNainiene, RasaCantos-Barreda, AnaNalbone, LucaCanuti, MartaNalon, ElenaCapaldo, AnnaNandini, SarmaCapela E Silva, FernandoNannarone, SaraCapillo, GioeleNanni Costa, LeonardoCapion, NynneNapoli, EttoreCapoccioni, FabrizioNara, AtsukiCapomaccio, StefanoNarat, VictorCappai, Maria GraziaNarayan, EdwardCapparè, PaoloNarute, PurushottamCappelletti, Giulio MarioNathanailides, CosmasCappelli, KatiaNava, GerardoCaprarulo, ValentinaNavas, LuigiCapucchio, Maria TeresaNazki, SalikCaputo, ValerioNdiaye, KalidouCarcangiu, VincenzoNeethirajan, SureshCardinali, IreneNeglia, GianlucaCardoso, Diercles FranciscoNekaris, AnneCaria, MariaNěmcová, LucieCarillo, FelicettaNeofitou, NikosCarini, Robert M.Nery, JoanaCarlstead, KathyNetherton, Christopher L.Carlucci, RobertoNeubauer, ViktoriaCarney, Hazel C.Neupane, MaheshCaroprese, MariangelaNg, Chen SiangCarotenuto, YleniaNguyen, TrieuCaro-Vadillo, AliciaNicholas, FrankCarr, MandiNicholas, RobinCarragher, JohnNicholson, SandraCarrasco, AdrianoNicodemus, MollyCarrera, MónicaNicosia, AldoCarretero, Miguel A.Niedziółka, RomanCarroll, GraceNiel, LeeCarter, AnneNielsen, BrianCarter, JenniferNielsen, Tina S.Carthy, TaraNielsen, Vance G.Caruso, GabriellaNiemiec, TomaszCaruso, GiuliaNiero, GiovanniCarvalho, António PauloNiewiadomska, ZuzannaCarvalho, PedroNii, TakahiroCasal, Jordi M.Nijman, VincentCasals, RamónNikinmaa, MikkoCasarrubea, MaurizioNikitkina, ElenaCasas, GloriaNiknafs, ShahramCasas, IsabelNiku, MikaelCascone, GiuseppeNilforooshan, Mohammad AliCastaldo, LucianaNinhaus-Silveira, AlexandreCastaneda, ClaudiaNinomiya, ShigeruCastejón-Riber, CristinaNishida, TakehiroCastelan Ortega, Octavio AlonsoNitulescu, MihaiCastellaro, Giorgio G.Nixon, Daniel W.Castillo Lopez, EzequiasNixon, JaneCastillo, CristinaNizza, AntoninoCastillo, PabloNkukwana, ThobelaCasto, Joseph M.Noce, AntoniaCastro, Fidel OvidioNocera, IreneCasu, SaraNogalska, AnnaCatala, AmélieNogalski, ZenonCatarina, StefanelloNogueira, TeresaCatone, GiuseppeNolasco-Soria, Héctor G.Cazapal-Monteiro, Cristiana FilipaNonacs, PeterCebulska, AleksandraNørgaard, Jan VærumCeccarelli, PieroNorman, TrevorCecchini, StefanoNormando, SimonaCeccobelli, SimoneNörnberg, José LaerteCeli, PietroNorouzitallab, ParisaCenariu, MihaiNorris, Stuart L.Cerasoli, IlariaNorton, ElaineCerda, ArtemiNotari, LorellaCerdà-Cuéllar, MartaNout-lomas, YvetteCerda-Reverter, Jose MiguelNovak, MelindaCerolini, SilviaNovoselec, JosipCerqueira, Aloysio M. F.Nowak, RaymondCerqueira, Joaquim Orlando LimaNowakiewicz, AnetaCerqueiro-Pequeño, JorgeNowakowicz-Dębek, BożenaCervantes, MiguelNowakowski, Jacek J.César, Aline Silva MelloNowicki, ArkadiuszCesarani, AlbertoNowosad, JoannaChadio, StellaNumberger, DanielaChae, Byung-JoNunes, Helder P.Chai, LilongNunome, MitsuoChaintoutis, Serafeim C.Nyman, AnnChakarov, NaydenO. Mgbere, OsaroChakraborty, TapasOanh, Nguyen CongChakravarthi, V. PraveenOba, AlexandreChalvatzi, SofiaOba, MasahitoChambers, ChristopherOber, Christopher P.Chang, Ching-FongOberbauer, Anita M.Chang, YuObremski, KazimierzChapman, NadineO’Brien, Justine K.Charmley, EdOchota, MałgorzataCharneca, Rui Miguel CarrachaOczkowicz, MariaCharvet, ClaudeOdierna, GaetanoChastant-Maillard, SylvieOem, Jae-KuChatziplis, DimitriosOgawa, ShinichiroChaucheyras-Durand, FrederiqueOgi, AsahiChavan, HemantkumarOguejiofor, ChikeChavatte-Palmer, PascaleOh, Sang-IkChedea, Veronica SandaOhmori, KeitaroChen, Ching-YiOishi, IsaoChen, ChongxiaoOjo, MercyChen, Jiann-ChuOkamoto, HitoshiChen, LianminOkuzawa, KoichiChen, Ming-JuOlagoke, OlusolaChen, Shuen-EiOldenbroek, KorChen, Tsung MingOleksa, AndrzejCheng, HengweiOliva, JessicaCheng, JianOliveira, GersonCheng, Vincent Yeong-HsiangOliveira, Karla Mychellyne CostaCheng, Wei NeeOliveira, Ronaldo LopesCheng, Yeong-HsiangOliveira, TeresaChenier, Tracey S.Oliveira-Filho, José P.Chiandetti, CinziaOliver, JoanneChicharro, DéborahOlivera, MarthaChimienti, MariannaOlives, Ana Martí-DeChincarini, MatteoOlivotto, IkeChládek, GustavOlmo, EttoreChmelíková, EvaOlson, Kenneth C.Cho, SohyunOlsson, AnnaChoi, Chun WhanOlukosi, Oluyinka A.Choi, In HagOlynk Widmar, Nicole J.Cholewińska, PaulinaO’Malley, Carly I.Chong, RogerOmatsu, TsutomuChou, Jen YunOmi, ToshinoriChourbaji, SabineOno, TakashiChow, BettyOnzima, RobertChow, Pizza Ka YeeOrczewska-Dudek, SylwiaChow, SeinenOrengo, JuanChristensen, Bruce W.Orihuela, AgustinChristensen, Janne WintherOrioli, ValerioChristensen, RachaelOrkusz, AgnieszkaChristian, SchulzeOrlowski, SaraChristodoulopoulos, GeorgiosOrnaghi, MarianaChu, ThinhOrosz, LászlóChulayo, Amanda YuccaOrtega, SofiaChun, Kwang-HoonOrtmeyer, Heidi K.Chur-Hansen, AnnaOrtuño, AnnaChwalibóg, AndréOrusa, RiccardoChwen, Loh TeckOssi, FedericoCiani, EliaOssiboff, Robert J.Ciani, FrancescaOster, MichaelCiccarelli, StefanoOsthoff, GarryCieslak, AdamOstović, MarioCieślak, JakubOtero, Pablo E.Cieślińska, AnnaOtten, WinfriedCilia, GiovanniOtto, Cynthia M.Ciliberti, Maria GiovannaOtto, JohnCinar, Mehmet UlasOttoboni, MatteoCincović, Marko R.Otwinowska-Mindur, AgnieszkaCinq-Mars, DanyOtzen, HenningCinque, CarloOuédraogo, DominiqueCiotola, FrancescaOuellet, VéroniqueCircella, ElenaOviedo-Rondón, Edgar OrlandoCitek, JindrichOvtchinnikov, IgorCitterio, CarloOwczarczak-Garstecka, SaraCizek, AloisOwens, David W.Claire, Wathes D.Ozkan, SezenClaridge, Andrew W.Pacchioli, Maria TeresaClaude, JulienPacífico, CátiaClavenzani, PaoloPadalino, BarbaraClayton, EdwardPadilla, DanielClegg, Simon R.Padilla, LorenaCleveland, Beth M.Page, ChadClifton, RachelPage, StephenCloutier, SylviePageat, PatrickClutton, EddiePage-Karjian, AnnieCocco, RaffaellaPahm, SarahCockrum, Rebecca R.Paim, Tiago Do PradoCocumelli, CristianoPaine, StuartCogger, NaomiPairis-Garcia, MoniqueCohen, ShariPalacios, VicenteColaço, Bruno JorgePalazzo, MarisaCole, KimberlyPalencia, JorgeCole, N. AndyPalin, Marie-FranceColeman, BobPalinski, RachelColeman, KristinePalladini, AlessandraColino-Rabanal, Victor J.Palladino, AntonioCollazo, Jaime A.Pallavicini, AlbertoCollivignarelli, FrancescoPalme, RupertColombo, MartinaPalmerini, MariaColominas-Ciuró, RogerPalomo Yagüe, AntonioColonna, Maria AntoniettaPaludo, Giane ReginaColpoys, JessicaPalus, KatarzynaColumbano, NicoloPan, Chieh-YuColussi, SilviaPanarello, GiuseppeComin, AriannaPanasiewicz, GrzegorzConan, AnnePandey, SumaliCondezo Castro, Gabriela NéridaPaolillo, MayraConnor, LauriePaolini, AlessandraConnor, MelaniePaolucci, MarinaConrady, BeatePapadomichelakis, GeorgeContalbrigo, LauraPapadopoulos, Elias G.Contiero, BarbaraPapadopoulos, GeorgiosContino, Erin K.Papadopoulos, SerafeimCook, BrianPapatsiros, Vassilios G.Cooke, Andrew S.Papazoglou, Lysimachos G.Coombe, JoannePapich, Mark G.Coombs, TamsinPapović, TamaraCooney, RosieParadhipta, Dimas Hand VidyaCoppola, Crista L.Paranhos Da Costa, Mateus José RodriguesCorassa, AndersonParenté, AlexisCorazzin, MircoParés-Casanova, Pere M.Corbee, Ronald JanParisi, FrancescaCorbera, Juan AlbertoParma, PietroCorbière, FabienParraguez, Víctor HugoCorda, AndreaParra-Olea, GabrielaCorino, CarloParrilla, InmaculadaCorletto, FedericoParrino, VincenzoCornale, PaoloParthasarathy, AnutthamanCorner-Thomas, Rene A.Paschino, PietroCorrea, FedericoPascoe, PeterCorreia, AlexandraPascual, MariamCorriero, AldoPasicka, EdytaCorsato Alvarenga, IsabellaPasolini, Maria PiaCort, JosePasquariello, RolandoCortes, OscarPassero, FelipeCortese, LauraPassler, ThomasCortes-Rodriguez, NandadeviPastore, NunziaCortez, PauloPastorino, PaoloCorti, ClaudiaPatarata, LuísCosby, DouglasPaterson, MandyCosentino, CarloPatience, JohnCossu, PieroPatil, AnandCosta, AngelaPatil, VeerupaxagoudaCosta, AnnamariaPatlar, BaharCosta, Giovanna LucreziaPaton, MichaelCosta, MónicaPatra, Amlan KumarCostas, BenjaminPatsikas, MichailCosti, ElenaPatuzzi, IlariaCostilla, RoyPauciullo, AlfredoCoustau, ChristinePaudel, SuryaCove, MichaelPaula, TilleyCowl, VeronicaPaulruds, Carl OskarCozzi, CristinaPausch, HubertCozzolino, RobertoPavani, Krishna ChaitanyaCrabtree, James R.Pavlata, LeosCraig, AllisonPawlina, KlaudiaCraig, JessicaPazos, Antonio JuanCrandell, KathleenPeaston, AnneCranston, LydiaPech Cervantes, Andres AlfredoCravana, CristinaPechova, AlenaCrean, Angela J.Pecka-Kiełb, EwaCrenshaw, ThomasPeckham, Gabriel D.Crépeaux, GuillemettePedernera Romano, MarianaCrespo, FranciscoPedraza Chaverri, JoséCretan, RemusPei, Kurtis Jai-ChyiCreutzinger, Katherine C.Peiretti, Pier GiorgioCribb, ThomasPeixoto, Maria JoãoCrisci, ElisaPeli, AngeloCrisi, Paolo EmidioPelttari, KaroliinaCrispin, SheilaPeña, EmyrCristarella, SantoPenaranda, IreneCroitor, RomanPenfold, LindaCronin, Katherine A.Penrith, Mary-LouiseCross, IsmaelPeralta-Sánchez, Juan ManuelCrovetto, Gianni MatteoPerazzi, AnnaCrowley, JamesPerec-Matysiak, AgnieszkaCrumière, Antonin J. J.Perego, RobertaCrump, AndrewPereira, Artur CamposoCubero Pablo, María JoséPereira, Jorge A.Cuchillo-Hilario, MarioPereira, Rosa Lino NetoCue, Roger I.Perera, NipunaCuervo, BelenPerez Ibarra, IreneCuevas-Ramos, GabrielPerez Jimenez, Jèsus MariaCui, YongdePerez Vendrell, Anna MariaCummins, Leo J.Perez, HectorCunha, MariaPérez, José AntonioCurl, AngelaPérez, José FranciscoCurtasu, Mihai VictorPérez, Luz M.Cussen, VictoriaPérez-González, JavierCutrignelli, Monica IsabellaPérez-Marcos, MaríaCwiklinski, KrystynaPérez-Martínez, ClaudiaCwynar, PrzemyslawPerez-Pe, RosauraCywinska, AnnaPeriago, Maria VictoriaCzech, AnnaPeris-Frau, PatriciaCzeglédi, LeventePerliński, PiotrCzerniejewski, PrzemysławPerotto, Humberto L.Czernik, MartaPerricone, VeraCzerwik-Marcinkowska, JoannaPerrucci, StefaniaCzopowicz, MichałPerry, ErinCzyż, KatarzynaPersson, YlvaCzyzewska-Dors, EwelinaPerzanowski, KajetanDa S. Cardoso, AbmaelPesti, Gene M.Da Silva Ávila, Carla LuizaPeterson, Karianne LievaartDa Silva Camargo, SandroPeterson, MichaelDa Silva Dias, Igor AlexandrePetow, StefanieDa Silva Junior, Diógenes CecilioPetracci, MassimilianoDa Silva Martins, Nelson RodrigoPetrera, FrancescaDa Silva Viana, GabrielPetri, ReneeDa Silva, Luís C. N.Petrini, JulianaDabbou, SihemPetrizzi, LucioDąbrowska, JoannaPetry, AmyDabrowski, RomanPetrželková, KláraDahlke, Flemming T.Pfaller, ChristianDahl-Pedersen, KirstinPhysick-Sheard, P. W.Dai, BoPiantino Ferreira, Antonio JoséDailey, RobertPiccioli Cappelli, FiorenzoDaisuke, KondohPiccione, GiuseppeDalla Costa, FilipePiccionello, Angela PalumboDalla Villa, PaoloPiccirillo, AlessandraDall’Aglio, CeciliaPiccolo, GiovanniDallago, GabrielPickova, JanaDallares, SaraPienaar, RonelDalloul, RamiPieramati, CamilloDalton, HillaryPierce, EllenDaly, KristianPierdon, MeghannDam Otten, NinaPierini, AlessioDam, Chinh Thi MyPieszka, MagdalenaDamasceno, Flavio AlvesPieszka, MarekDamaziak, KrzysztofPietroluongo, GuidoDamiaans, BertPietrosemoli, SilvanaD’Amico, MarcelloPietruszka, ArkadiuszDamiran, DaalkhaijavPiles, MiriamDanchin-Burge, CoraliePilla, RachelDandrieux, JulienPilli, NageswaraDänicke, SvenPiltz, JohnDaniele, AuroraPinares Patino, CesarD’Aniello, BiagioPinart, ElisabethDaniels, SimonPiñeiro, CarlosDaros, Ruan R.Pinna Parpaglia, Maria L.Darre, Michael J.Pinotti, LucianoDarwich, LailaPinou, TheodoraDashti, AlejandroPinto, Andreia L.Dastjerdi, AkbarPiórkowska, KatarzynaDaszkiewicz, TomaszPiotti, PatriziaDavail, BlandinePiquer, OlgaDavies, MichaelPirolo, MattiaDavies, Peers L.Piscopo, MarinaDavies, SimonPiskunov, Alexey K.Davis, Adam J.Pitino, RosarioDavis, ChelseaPiwczyński, DariuszDavis, ElizabethPiwowarski, JakubDavoli, FrancescaPlachá, IvetaDawson, JessicaPlanas Oliver, MiquelDay, JohnPlascencia, AlejandroDayem, Ahmed AbdalPlavec, TanjaDe Andres Cara, Damian F.Pławińska-Czarnak, JoannaDe Araújo Oliveira, Chiara AlbanoPlush, Kate J.De Araújo, José Pedro PintoPodgórska, KatarzynaDe Biase, DavidePodnar Lešić, MartinaDe Boni, AnnalisaPoessel, Sharon A.De Bonis, SalvatorePogorzelska-Nowicka, EwelinaDe Briyne, NancyPohlin, FriederikeDe Camargo, Gregório Miguel FerreiraPoirier, AliceDe Carvalho, Gleidson Giordano PintoPoirier, CollineDe Castro, Ana Catarina VieiraPol, FrançoiseDe Clercq, DominiquePolak, GrażynaDe Cramer, Kurt G. M.Polak-Śliwińska, MagdalenaDe Felice, ElenaPolasik, DanielDe Figueirêdo Freitas, Vicente JoséPoley, Lucy G.De Gregorio, BrettPolidori, PaoloDe La Fuente Crespo, Luis FernandoPolinas, MartaDe La Fuente, GabrielPolizel, Daniel M.De La Vega, P. T. MartínPollard, DanicaDe Lagarde, MaudPollitt, ChrisDe Liberato, ClaudioPolo, JavierDe Lima Santos, RenatoPombo, AnaDe Lima, Valéria Marçal FelixPomerantz, OriDe Luca, SilvioPomianowski, AndrzejDe Medeiros, Sérgio RaposoPomilio, FrancescoDe Mol, Rudi M.Pomorska-Mól, MałgorzataDe Moraes, Francisca PereiraPongrácz, PéterDe Mori, BarbaraPonte, GiovannaDe Oliveira, Cláudio Marcelo GonçalvesPontigo, Juan PabloDe Olivera, MariaPoock, ScottDe Pasquale, CairstyPoole, Joyce H. P.De Rensis, FabioPoole, RebeccaDe Santis, FrancescaPoole, Toni L.De Segura, Ignacio GomezPopescu, SilvanaDe Souza, Fernando NogueiraPorr, SheaDe Spiegelaere, WardPortier, Karine GenevieveDe Waal, TheoPorto, SimonaDe Zani, DonatellaPosautz, AnnikaDeblitz, ClausPosbergh, ChristianDec, MartaPoser, HelenDecaro, NicolaPotter, KatieDechant, ReinhardPoulicek, Mathieu E. A.Deflorio, MichelePoulopoulou, IoannaDegraves, FredPourlis, ArisDel Curto, TimPovilitis, TonyDel Pino, Francisco Augusto BurkertPowell, LaurenDel Pozo-Acebo, LorenaPoyun, TengDel Prete, ChiaraPrada, JustinaDel Valle, Tiago AntonioPradhan, PrajalDelalande, Jean MariePrado, Ivanor Nunes DoDelbes, CélinePrado, María RodríguezDelerue, FabienPrakas, PetrasDelgado Bermejo, Juan VicentePramual, PairotDelgado, Juan VicentePrasun, DuttaDelghandi, MohammadPrenda, JoséDelia, LacastaPrescott, MarkDell, Andrew C.Přibyl, JosefDellamaria, DeboraPřibylová, LucieDell’Anno, MatteoPrieto, AlbertoDellinger, MyannaPrins, Jan-BasDelom, FrédéricPriyashantha, HasithaDeng, YunyanProbo, MonicaDeng, ZhaojuPróchniak, TomaszDenise, HebesbergerProchowska, SylwiaDenzin, NicolaiProla, LivianaDerks, Martijn F. L.Proszkowiec-Weglarz, MonikaDervishi, EldaPrpić, ZvonimirDeters, Erin LeaPruisscher, PeterDettori, Maria LuisaPrus, PiotrDevoy-Keegan, JasonPruszynska-Oszmalek, EwaDevriendt, NausikaaPryimenko, NathalieDeyneko, IgorPrzybylski, WiesławDhanasiri, AnushaPszczola, MarcinDhar, ArunPtacek, MartinDi Bella, SantinaPuccinelli, MartinaDi Bello, AntonioPuggioni, AntonellaDi Bene, ClaudiaPugliese, MichelaDi Cerbo, AlessandroPujol, Flor H.Di Costanzo, AlfredoPukazhenthi, Budhan S.Di Emidio, GiovannaPulcini, DomitillaDi Francesco, Cristina EsmeraldaPulido, Rubén GuillermoDi Loria, AntonioPurcell, MaureenDi Martino, GuidoPurdy, Phil H.Di Micco, LuigiPutman, Ashley K.Di Nardo, GiovannaPutz, Austin M.Di Palma, AntonellaPutz, EllieDi Paola, RosannaQazi, Izhar HyderDi Pasquale, JorgelinaQian, XuejunDi Persio, SaraQuaglio, FrancescoDi Stasio, LilianaQuandt, JaneDi Tommaso, MorenaQuaresma, MiguelDiagne, ChristopheQuartuccio, MarcoDiakou, AnastasiaQudsieh, RashaDiana, AlessiaQueiros, JoãoDias Marques, Maria IsabelQueiróz, Luzia HelenaDias Schleder, DelanoQuesta, MariaDíaz Gaona, CiprianoQuinn, NiamhDíaz López, OliverQuiñones, JohnDiaz-Bonilla, EugenioQuinonez, Fausto ValenzuelaDíaz-Sánchez, Adrian AlbertoQuintana, Álvaro RafaelDickinson, EdwinQuintas, HelderDickson, JamesRachwald, AlekDidkowska, AnnaRackwitz, ReikoDiéguez, Francisco JavierRadlinsky, Mary Ann G.Dierick, EvelienRaedts, Peter J. M.Dietz, Maurine W.Raekallio, MarjaDigman, MatthewRafales, Cristina BonastreDiioia, MarinaRaffrenato, EmilianoDindot, Scott V.Ragan, IzabelaD’Ingeo, SerenellaRagkos, AthanasiosDiogo, PatriciaRagni, MarcoDistl, OttmarRahman, Masmudur M.Dittmer, KerenRai, NavneetDixon, LauraRaidan, Fernanda S. S.Dixon, RobertRairat, TirawatDjikanović, VesnaRamachandran, ReshmaDobos, RobinRamalho-Santos, JoãoDobrowolski, PiotrRamal-Sanchez, MarinaDocea, Anca OanaRambozzi, LuisaDoceul, VirginieRamenofsky, MarilynDodd, SarahRamilan, ThiagarajahDoğan, SalihRamirez, AlejandroDoherr, MarcusRamírez, AlfredoDomeniconi, Raquel FantinRamirez, BrettDomínguez Rebolledo, Álvaro EfrénRamírez-Avilés, LuisDomínguez, JesúsRamirez-Bribiesca, J. EfrenDomino, MalgorzataRamírez-Mella, MónicaDonadelli, Renan AntunesRamiro Rodriguez, ManuelDonnelly, Callum GeorgeRamos, Eric A.Dörgeloh, WernerRancilio, Nicholas J.Dors, ArkadiuszRand, JacquieDos Santos, Fábio A. AbadeRandazzo, BasilioDos Santos, Geraldo TadeuRandhawa, ImtiazDos Santos, Regiane RodriguesRandle, HayleyDos Santos, Tatiana CarlescoRando, AndreaDotas, VassiliosRanninger, ElisabethDotsev, ArsenRant, WitoldDoty, Jeffrey B.Raoult, CamilleDoughty, AmandaRapetti, LucaDovč, AlenkaRaskovic, BozidarDowning, JeffreyRasmussen, Morten DamDoyle, EmmaRasmussen, Sophie LundDoyle, HesterRast, LuziaDrażbo, Aleksandra AlicjaRatnayake, ChamindaDrevet, Joel R.Rato, Luís Pedro FerreiraDriessen, BertRaudsepp, TerjeDritsas, EliasRavera, IvanD’Souza, DarrylRavetto Enri, SimoneDu, XiaoyongRavindran, VelmuruguDuarte, MargaridaRawat, AnoopDubey, RaghvendraRayburn, Edward B.Duda, MałgorzataRaza, Sayed Haidar AbbasDuenk, PascalRazzuoli, ElisabettaDufrasne, IsabelleRead, JohnDugdale, Alexandra H. A.Read, RachelDuggan, JenniferReal Valcárcel, FernandoDulisz, BeataRebucci, RaffaellaDumètre, AurélienRedaelli, VeronicaDuncan, Luke MangalisoReed, KristanDundas, Shannon J.Reese, Laura A.Dunisławska, AleksandraReeve, NigelDunner, SusanaRegal, PatriciaDuplessis, MélissaReggi, SerenaDurán, Manuel NovalesRego, João Paulo A.Durland, EvanRegolin, LuciaDuscher, Georg GerhardRehbein, SteffenDvoretsky, Alexander G.Reid, ScottDvoretsky, VladimirReiko, NagataDybus, AndrzejReimert, HennyDyson, SueReis, Joana Da CostaDzidic, AlenReis, Ricardo AndradeDzięgielewska, MagdalenaRekiel, AnnaDziekońska, AnnaRelling, Alejandro E.Dzierzęcka, MałgorzataRemesar, SusanaDżugan, MałgorzataRemot, AudeEbensperger, GermánRempel, Lea A.Ebertz, PeterRenna, ManuelaEbinghaus, AsjaRenner, Swen C.Eddison, JohnRensi, NicolòEde, ThomasRenu, SankarEdwards, Gaylen L.Repetto, OmbrettaEdwards, Katie L.Requena, FranciscoEdwards, Sandra A.Resconi, Virginia CeliaEgberink, HermanReséndiz, EduardoEgerszegi, IstvánRestrepo, GiovanniEichenberger, Ramon MarcRestucci, BrunellaEik, Lars OlavRevilla, IsabelEinspanier, RalfReynolds, Michelle H.Eira, CatarinaRibeiro De Souza Da Cunha, Maria De LourdesEl Nagar, Ayman Gamal FawzyRibeiro-Filho, Henrique M. N.Elbers, Armin R. W.Ricci, PasqualeEl-Deeb, WaelRichards, NgaioElkhoraibi, CarineRichardson, Jill L.Ellen, KienzleRickard, Jessica PaigeEllerbrock, RobynRickards, KarenElliott, JonathanRico, J. EduardoEllis, Andrea DorotheaRiddick, Eric WellingtonEllis, BrianRidler, Anne L.Elmahallawy, EhabRidpath, JuliaElmi, AlbertoRidrigo, AnaElnesr, ShaabanRiegert, JanElolimy, Ahmed A.Rifici, ClaudiaElsohaby, IbrahimRigaill, LucieElvang Jensen, HenrikRiley, ChristopherElvers, GregRiley, Lisa M.Embregts, CarmenRiley, SophieEndres, MarciaRincon, Angela M.Endres, ThomasRinder, MonikaEngelhardt, SachaRinnovati, RiccardoEngelken, TerryRipoll, GuillermoEpperson, WilliamRitter, CarolineErickson, PeterRiva, FedericaErnest Blevins, JamesRivera Del Álamo, Maria MontserratErnst, Ulrich RainerRivero Viera, JordanaEscobar, SebastianRoana, JaniraEspinosa, CharmaineRoberto Da Costa, R. PlacidoEspinosa, VíctorRoberts, JulieEspírito Santo, ChristopheRoberts, Juliet R.Esposito, GiuliaRobertson, Sheilah AnnEsposito, LuigiRobertson, SusanEssner, AnnRobinson, CharlotteEsteban-Blanco, CristinaRobinson, KelsyEstensoro, ItziarRobles, MorganeEstrada, Jose L.Roca-González, José LuisEstrada-Angulo, AlfredoRocchigiani, GuidoEszter, Hubainé TóthRocha Faustino, Ana IsabelEto, Silas FernandesRocha, Gabriel CiprianoEvans, Jacquelyn M.Rocha, Luiene M.Evans, KateRoche, StevenEvans, RhysRode-Margono, JohannaEwa, Sosnówka-CzajkaRodero Serrano, EvangelinaExbrayat, Jean-MarieRodger, JohnEzquerra Calvo, Luis J.Rodrigo Mocholi, DiegoFabbri, GiorgiaRodrigues Da Costa, MariaFabbri, Maria ChiaraRodrigues Pires Júnior, OsmindoFabia, FonRodrigues, CarlindoFablet, ChristelleRodríguez Alvarez, LleretnyFàbrega-Romans, EmmaRodriguez, KerriFabrizio, MauroRodríguez, MartínFaccia, MicheleRodríguez, PedroFaccini, SilviaRodríguez, Virginia GaminoFadok, ValerieRogers, Chris W.Fadul-Pacheco, LilianaRogers, JeffreyFages, AntoineRogers, SuzanneFajfer, MonikaRogiewicz, AnnaFaleiros, RafaelRohaim, MohammedFalta, DanielRojo, Maria AngelesFantinati, MarcoRoman, MichałFarmer-Dougan, ValeriRomano, NicholasFarnworth, MarkRomanov, Michael N.Fasina, FolorunsoRomański, MichałFassani, Édison JoséRombenso, Artur NishiokaFatone, GerardoRomeiro Fernandes Chagas, CarolinaFattorini, NiccolòRomero, JaimeFaustini, MassimoRomero-Aguirregomezcorta, JonFaustino, AnaRomito, GiovanniFavre, David S.Romussi, StefanoFawzy, Ahmad MahmoudRondina, DavideFaye, BernardRoperto, FrancoFazio, EsterinaRopka-Molik, KatarzynaFazio, FrancescoRoque, André Luiz RodriguesFeddern, VivianRoques, JonathanFeito, JavierRoque-Torres, Gina DeliaFelix, AnandaRos Berruezo, GasparFenner, KateRosa Salva, OrsolaFerenc, PajorRosa, BrielleFerlazzo, AdrianaRosado, BelénFerlazzo, AlidaRose, PaulFernandes, Luis Guilherme VirgílioRosenthal, BenjaminFernandez, IgnacioRoss, SteveFernández, MiguelRossi, FilippoFernández-Luqueño, FabiánRossi, LucaFernandez-Novo, AitorRota, AdaFernández-Robledo, José AntonioRotllant, Guiomar E.Fernando Ivan Flores, PerezRoughan, Johnny V.Fernando, DeepaniRoux, Delphine LeFernando, FerreiraRowan, AndrewFerosekhan, ShajahanRowe, AnthonyFerra, BartłomiejRoy, BidishaFerramosca, AlessandraRoy, JérômeFerrari, AliceRozsa, LajosFerraz, Guilherme De CamargoRuberto, TommasoFerraz, Marcia A. M. M.Rubio San Millan, Luis ÁngelFerraz, PatriciaRubio, André V.Ferre, IgnacioRuckstuhl, Kathreen E.Ferreira, Ana CristinaRuczizka, UrsulaFerreira, DavidRudeck, JulianeFerreira, FernandoRueca, FabrizioFerreira, FranciscoRuggeri, ElenaFerreira, MarceloRuiz De Ybáñez, María RocíoFerreira, SamRuiz Riquelme, Alejandro IvánFerreira, Vitor Hugo BessaRuiz, HéctorFerreras, PabloRuiz-Albarrán, MiguelFerretti, GiannaRupik, WeronikaFerris, Conrad P. W.Russell, James R.Ferro, SilviaRusso, CarloFertő, ImreRustad, TuridFey, DariuszRustas, Bengt-OveFichi, GianlucaRutberg, Allen T.Fielitz, JensRuud, Lars ErikFigliola, LuciaRuviaro, Clandio FavariniFigueroa, JaimeRužauskas, ModestasFiliciotto, FrancescoRybska, MartaFinzi, AlbertoRychel, JessicaFiore, EnricoRyczek, NataliaFioretti, AlessandroRząsa, AnnaFiorito, GrazianoSaastamoinen, MarkkuFiorucci, LetiziaSabino, MarcellaFischer-Tenhagen, CarolaSacchetti, PatriziaFlach, LauraSacchini, SimonaFlis, MarianSach, FionaFlisikowski, KrzysztofSachin, SharmaFlorín-Christensen, MonicaSadkowski, TomaszFlorowski, TomaszSaelao, PerotFoglia Manzillo, ValentinaSáenz, LeonardoFondevila, ManuelSaettone, VittorioFong, KarenSaha, SudebFonseca, PabloSaito, ShouichiroFonseca, Rui PedroSaitoh, KenjiFonseca-Alves, Carlos EduardoSakomura, Nilva KazueFontes, PedroSakowski, TomaszForder, Rebecca E. A.Salamatin, RusłanForeman, Anne M.Salas, MarinaForis, BorbalaSalazar-Villanea, SergioForkman, BjörnSaleh, Mayra A. D.Formenti, NicolettaSaliu, Eva-MariaForte, ClaudioSalmons, BrianFortina, RiccardoSalvarina, IoannaFosgate, GeoffSalvatori, ValeriaFoskolos, AndreasSalvin, HannahFoster, Derek M.Samaras, AthanasiosFotedar, RaviSamardžija, MarkoFoucras, GillesSamarin, Azin MohagheghiFox, Michael W.Samartzi, FotiniFoyle, LeoSamet, LaurenFraile, LorenzoSamiec, MarcinFraley, Gregory S.Sampaio, RafaelFranchini, DeliaSamuel, RyanFranciosini, MariaSan Andres, ManuelFrancisco-Morcillo, JavierSánchez, Julio ÁlvarezFranco, Gumercindo LorianoSánchez, Ramón AriasFranczak, AnitaSánchez-Ajofrín, IreneFrank, Eduardo N.Sanchez-Cordero, Victor ManuelFrank, KrisztiánSánchez-Escalante, ArmidaFrankel, Theresa L.Sánchez-Margallo, Francisco MiguelFrant, MaciejSanchis, JaimeFranzo, GiovanniSandersen, Charlotte F.Franzoi, MarcoSandgren, EricFranzoni, GiuliaSandmann, MichaelFraser, Thomas William KennethSandøe, PeterFreeman, MarianneSandor, ZsuzsannaFregeneda-Grandes, Juan-MiguelSandoval Castro, Carlos AlfredoFrei, BarbaraSaneyasu, TakaokiFreire, João Pedro BengalaSanna Passino, EraldoFrey, CarolineSano, TadashiFrias, LiesbethSant’Anna, Aline CristinaFriedenberg, Nicholas A.Santín, MónicaFriend, TedSantonicola, SerenaFriendship, RobertSantos López, Eva M.Frizzi, FilippoSantos, Jesus A.Frosth, SarahSantos, Virgínia Alice Cruz DosFry, Lindsay M.Santos-Gomes, GabrielaFrynta, DanielSantos-Jimenez, ZurisadayFuchs, ClemensSapierzyński, RafałFuchs-Baumgartinger, AndreaSapountzis, PanagiotisFugazza, ClaudiaSaqui-Salces, MilenaFukami, KimioSaraiva, JoaoFukuma, Naoki M.Saraz, Jairo A. O.Funahashi, HiroakiSarli, GiuseppeFurstenau, Tara N.Sarmiento García, AinhoaFurtado, TamzinSarmiento-Franco, LuisFuruita, HirofumiSaroglia, MarcoFusaro, IsaSarriés, María VictoriaFuseini, AwalSartori, CristinaFusi, FrancescaSatué, KatiuskaFusi, JasmineSavic, RadomirFusté-Forné, FrancescSavini, FilippoFyfe, JohnSavoca, SerenaGäbel, GottholdSavvas, IoannisGabler, ChristophSawicka-Zugaj, WiolettaGabriël, SarahSawosz, EwaGácsi, MártaSayedahmed, EkramyGagaoua, MohammedScandurra, AnnaGajardo, GonzaloScanes, Colin G.Galarce, NicolásScanziani, EugenioGalatos, Apostolos D.Schaefer, Allan L.Galfano, GiovanniSchapiro, SteveGalkina, Svetlana A.Scharf, ValeryGallagher, Meurig T.Schelling, EstherGallina, SoniaScherer, UlrikeGallo, CarmenSchiariti, AgustínGallo, LuigiSchiavetti, AlexandreGama, AdelinaSchiavo, GiuseppinaGamperl, A. KurtSchiavone, AchilleGandini, GustavoSchlagloth, RolfGandolfi, FulvioSchmid, Jeffrey RobertGantner, MagdalenaSchmid, MarkusGao, ChunqiSchmidt, VolkerGarbossa, Cesar Augusto PospissilSchmitt, OcéaneGarces Narro, CarlosSchmucker, SonjaGarcia Ispierto, IrinaSchoiswohl, JuliaGarcia Pardo, Maria De La LuzScholtens, MeganGarcia, Andre Luiz SeccattoScholz, ArminGarcía, Antón R.Schoon, AdeeGarcía, GracielaSchött, UlfGarcia, Lyda G.Schreiter, RubenGarcía-Álvarez, OlgaSchroeder, KatyGarcía-Belenguer, SylviaSchröter, IrisGarcia-Dominguez, XimoSchroyen, MartineGarcia-Estañ, Luis PerezSchuberth, Hans J.García-Gasca, Silvia AlejandraSchug, Malcolm D.García-Germán, SolSchulte, Bruce AlexanderGarcia-Ispierto, IrinaSchulte, Lisa M.Garcia-Perez, Ana L.Schulz, CarstenGarcía-Rebollar, PalomaSchusser, Gerald FritzGarcía-Rodríguez, AserSchutz, MichaelGarcia-Souto, DanielSchwabe, KerstinGardón, Juan CarlosSchwarz, TobiasGargano, ValeriaSchwarz, TomaszGarmyn, AndreaSchwarzbacherová, VieraGarnsworthy, PhilSchwarzer, AngelaGarry, Mary G.Schwean-Lardner, Karen V.Garstang, MichaelScillitani, GiovanniGasa, JosepScobie, DavidGaspa, GiustinoScobie, LindaGastal, Gustavo Desire AntunesScopa, ChiaraGatel, LaureScott, EricaGates, RichardScott, Harvey MorganGatto, EliaScott, IanGautier, CamilleSeabra, Sofia G.Gavaia, PauloSearcy, Christopher A.Gawor, JakubSeeber, Peter A.Gaworski, MarekSeeth, Martin ThoGaxiola-Cortes, Martha GabrielaSegat, Hecson JesserGazzonis, Alessia LiberaSegura-Plaza, JoséGeary, TomSeidavi, AlirezaGeddes, Rebecca F.Sell-Kubiak, EwaGekenidis, Maria-TheresiaSeltmann, Martin W.Gennari, Solange MariaSelvaggi, MariaGenz, JanetSembratowicz, IwonaGeraldo, Ana C. A. Pereira De MiraSemik-Gurgul, EwelinaGermon, PierreSenczuk, GabrieleGernand, ErhardSeniczak, AnnaGerner, WilhelmSeo, JakyeomGershoni, MoranSeoane, PedroGervasoni, DamienSeppa, PerttuGesek, MichałSepulveda, NéstorGetchell, RodmanSerão, Nick V.Ghassemi Nejad, JalilSergiel, AgnieszkaGherman, Calin MirceaSerra, Cláudia R.Ghosh, SharmilaSerra-Cobo, JordiGiadinis, Nektarios D.Serrano, EmmanuelGiannetto, AlessiaSerrapica, FrancescoGiannetto, ClaudiaSerres, ConsueloGiannico, FrancescoSeshima, FumiGiansanti, DanieleSespere Oliveira, MaryaneGiantsis, Ioannis A.Sessions-Bresnahan, Dawn R.Giaquinto, Percília CardosoSeung-Hun, LeeGieryńska, MałgorzataSevi, AgostinoGieseke, DanielSevilla-Navarro, SandraGifford, Cody L.Sewint, NancyGil, EricSfacteria, AlessandraGil, Maria AntoniaSforzi, AndreaGiljov, AndreySgoifo Rossi, Carlo AngeloGilkerson, JamesShah, VrutantGille, LindeShakeri, MajidGiller, KatrinShakya, Akhalesh KumarGil-Sánchez, José MaríaShanmugasundaram, RevathiGimeno, MarinaSharma, ArvindGinés, Rafael RuizSharma, KavitaGioacchini, GiorgiaSharma, SandeepGioia, GloriaSharp, Claire R.Giotto, Francine M.Shaver, Randy D.Giovambattista, GuillermoShaw, AndrewGiráldez, Francisco JavierShearer, Jan KeithGiri, Sib SankarSheeran, LoriGirondot, MarcShelomi, MatanGisbert, EnricShen, GuofangGisondi, SilviaShepherd, MeganGispert, MarinaShi, HaoyuGiuliotti, LorellaShieh, Bao-SenGivisiez, Patricia Emilia NavesShigeno, ShuichiGkafas, GeorgiosShih, Hao YuGlaria, Idoia E.Shimabukuro, MasayaGleerup, Karina B.Shin, Dong-HyunGlenk, Lisa MariaShinozuka, YasunoriGluck, JohnShiraishi, Jun-ichiGmel, Annik ImogenShirak, AndreyGnat, SebastianShusuke, SatoGobello, CristinaSickinger, MarleneGodyń, DorotaSicuro, BenedettoGodzińska, Ewa JoannaSiddiqui, ArifGoerlich-Jansson, Vivian C.Sidiropoulou, ErasmiaGoetsch, Arthur LouisSiemieniuch, Marta J.Goff, Jesse P.Siena, GiuliaGołębiewska, BarbaraSierra, MatildeGoleman, MałgorzataSignal, TaniaGollmann, GünterSilacci, PaoloGolomazou, EleniSilva De Oliveira, JulianaGómez Álvarez, Constanza B.Silva, Alexandre R.Gomez Carpio, Mayra M.Silva, EdianeGomez, Ernesto A.Silva, EdsonGómez-Arrue, JavierSilva, FabyanoGomez-Piñero, EnriqueSilva, Josineudson Augusto Ii VasconcelosGomis, JesúsSilva, LuizGonçalves De Freitas, ElianeSilva, Nathalia Cristina CironeGoncalves, MartaSilva, Rodrigo Otávio SilveiraGonçalves, PedroSilva, Severiano R.Gonzaga Neto, SeverinoSilva, Vinícius PimentelGonzales, Jose L.Silvestri, GiuseppeGonzalez Montana, Jose RamiroSimeoni, FrancescoGonzalez Prendes, RaynerSimitzis, PanagiotisGonzalez, John MichaelSimm, GeoffGonzalez, ManuelSimões, João Carlos CaetanoGonzalez, RichardSimonik, OndrejGonzalez-Alvarez, MartaSinger, EllenGonzález-Barrio, DavidSingh, AmitGonzalez-Bulnes, AntonioSingh, BhupinderGonzález-Córdova, Aarón F.Singh, MiniGonzález-Cortazar, ManasésSingh, PreetGonzález-Doncel, MiguelSingh, YazavinderGonzález-Martín, Juan V.Singletary, MelissaGonzález-Martínez, ÁngelaSinsch, UlrichGonzález-Montaña, José RamiroSinski, Jennifer B.González-Ramírez, Mónica TeresaSipka, AnjaGonzález-Redondo, PedroSirtori, FrancescoGonzález-Ríos, HumbertoSitkowska, BeataGonzalez-Vicente, AgustinSitthicharoenchai, PanchanGood, Deborah J.Skarzynski, Dariusz JanGood, Sara V.Skibins, JeffreyGoodla, LavanyaSkiepko, NataliaGoodwin, WendySkliros, DimitriosGoossens, EvySkok, JankoGopegui, Rafael R.Skomial, JacekGordon, StuartSkoracki, MaciejGore, Mauvis A.Skřivanová, EvaGorman, KatieSkrlep, MartinGórski, KrzysztofSkrzyszowska, MariaGospodarek, JaninaSlater, MargaretGossman, ToniSlawinska, AnnaGoto, TatsuhikoSlotta-Bachmayr, LeopoldGöttert, ThomasSluder, Ann E.Gottschalk, ThomasSmaldone, GiorgioGougoulis, Dimitris A.Śmiałek, MarcinGoyache, FélixSmiley, Peter C.Goździewska-Harłajczuk, KarolinaSmith, Donald B.Grabner, Daniel S.Smith, Geoffrey R.Grabowski, TomaszSmith, GrahamGracia-Calvo, LuisSmith, Joe S.Graczyk, RadomirSmith, RobGradela, AdrianaSmith, Stephen A.Gradinaru, Andrei CristianSmith, Todd G.Graham, Melanie L.Smith, ZacharyGrahofer, AlexanderSneddon, LynneGraïc, Jean-MarieSnowdon, CharlesGrainger, MatthewSobek, ZbigniewGralak, Mikołaj AntoniSobiech, PrzemysławGranatosky, Michael C.Sobolewski, JarosławGrande Tovar, Carlos DavidSobotka, WiesławGrandin, TempleSocha, MagdalenaGraphodatsky, AlexanderSoglia, DomingaGrau, CarlosSogliani, DavideGray, AlisonSohn, Sea HwanGray, CarolSokolowski, MichelGreeff, JohanSoler Valls, Ana JosefaGreen, JennahSoler, María DoloresGreenberg, NeilSolka, MagdalenaGreene, ElizabethSondgeroth, KerryGreenwood, Alex D.Song, TongxingGreer, AlanSongsasen, NucharinGreggor, Alison LindaSońta, MarcinGreguła-Kania, MonikaSossidou, EvangeliaGreiman, StephenSossidou, Evangelia N.Greiner, LauraSoulsbury, Carl D.Grela, Eugeniusz RyszardSoumeh, Elham AssadiGresner, NinaSouthard, TeresaGrieco, ValeriaSouza-Fabjan, Joanna MariaGriffen, GillySoyeurt, HélèneGriffin, Taylor ChastainSpadavecchia, ClaudiaGrilli, GuidoSpadola, FilippoGrimm, HerwigSpanakis, MariosGrinde, AlexisSpeight, NatashaGrippi, FrancescaSpencer, TerryGrolli, StefanoSpengler Neff, AnetGroth, DavidSperone, EmilioGrove-White, DaiSperoni, MarisannaGruen, Margaret E.Spindler, BirgitGrunberg, Rita L.Spinella, GiuseppeGruszczyńska, JoannaSpinu, MarinaGruszecki, TomaszSponenberg, PhillipGryś, SławomirSpranghers, ThomasGryz, JakubSprícigo, José Felipe WarmlingGrzegorzewski, WaldemarSquire, Sylvia AfriyieGuan, JiewenSserwadda, MartinGuarino, CassandraStachurska, AnnaGuedes, SofiaStadnyk, AndrewGuenther, Katja M.Stadtlander, TimoGuerreiro, Pedro MiguelStahl, Chad H.Guest, Deborah JaneStaikou, AlexandraGugjoo, Mudasir BashirStalder, Kenneth J.Gugołek, AndrzejStangaferro, MatiasGuimarães, Simone E. F.Stangierski, JerzyGuinguina, AbdulaiStanley, DanaGulyaeva, MarinaStannard, Hayley J.Gumułka, MałgorzataStarling, Melissa J.Gurgul, ArturStayton, TristanGurtler, VolkerStefani, AnnalisaGutierre, SilviaStefania Longoni, SilviaGutierrez, JuanStefaniuk-Szmukier, MonikaGutierrez-Blanco, EduardoStefanović, MilomirGutiérrez-Chávez, Abner JosuéStefánsdóttir, GudrunGutiérrez-Gil, BeatrizStefanski, TomaszGutkowska, KrystynaSteffen, DavidGuzhva, OleksiyStein, Hans H.Guzmán, José LuisStein, TorsteinHachero, IsmaelSteinberg, Eliana RuthHać-Szymańczuk, ElżbietaSteinman, AmirHadley, PhilStella, JudithHaest, BirgenStellato, AnastasiaHäger, ChristineStenzel, Tomasz A.Hager-Theodorides, AriadneStephan, WildeusHagiya, KoichiStephens, NahiidHairgrove, ThomasStephens, PhilipHalas, VeronikaStępniowska, AnnaHales, JanniSter, CélineHall, EdwardSteri, RobertoHall, Emily J.Stetina, Birgit U.Hall, GeoffreySteuer, Ashley E.Hall, KatieStiller, JosefinHall, SophieStone, JoshuaHalle, IngridStout, Alison E.Hametner, KatharinaStracke, JennyHamilton, LindsayStrappini, AnaHamilton-West, ChristopherStraticò, PaolaHamon, MorganStreck, André FelipeHandler, JohannesStringhini, José HenriqueHannan, Md. AbdulStrong, AllanHanne, HansenStrube, Mikael LenzHans, MarkStrychalski, JanuszHanuš, OtoStrydom, Phillip E.Hanzen, ChristianStuart, BryanHao, HaihongStumpff, FriederikeHaraf, GabrielaSu, HuaweiHarms, CraigSuchocki, TomaszHarrison, TaraSuckow, Mark A.Hart, Lynette A.Sukhodolskaya, RaisaHart, SteveSulas, LeonardoHartstone-Rose, AdamSullivan, RyanHarvey, Andrea M.Sultana, HalimaHarvey, John W.Summer, AndreaHarvey, Kelsey Margaret SchubachSundar, SathishHasegawa, MakotoSurai, Peter F.Hashem, NesreenSuresh, VinodHashimoto, KoichiSutherland, IanHaskell, MarieSutton, Gila AbellsHaspeslagh, MaartenSuzuki, HiroshiHassen, AbubekerSuzuki, YutakaHässig, MichaelSwaggerty, ChristinaHaubenhofer, DoritSwerczek, ThomasHaubro Andersen, PiaŚwiątkiewicz, MałgorzataHaussler, KevinŚwitoński, MarekHawes, PhilippaSylla, LakamyHay, El HamidiSylvie, Chastant-MaillardHayashi, YoshiakiSymeonidou, IsaiaHayes, Morgan D.Szczepkowska, AleksandraHazel, Susan J.Szczerba-Turek, AnnaHedhammar, Åke A.Szczurek, WitoldHeiderstadt, KathleenSzeleszczuk, ŁukaszHeidor, RenatoSzendrő, ZsoltHeilen, Laura B.Szentiványi, TamaraHein, NilsSzóstek-Mioduchowska, AnnaHeinemann, Marcos BryanSzuba-trznadel, AnnaHeinen, JoelSzulc, KarolinaHeins, BradleySzymańczyk, Sylwia EdytaHeizmann, VeronikaSzymandera-Buszka, KrystynaHejcmanova, PavlaSzymanska, MagdalenaHeldbjerg, HenningSzymańska-Czerwińska, MonikaHeleski, CamieSzyndler-Nędza, MagdalenaHeller, E. DanTabler, TomHeller, JosephTaciak, MarcinHempel, SabrinaTadich, TamaraHemsworth, Lauren M.Taechangam, NopmaneeHendrickson, DeanTafuri, SimonaHendriks, Wouter H.Tajchman, KatarzynaHengjia, NiTajima, MotoshiHenkanaththegedara, SujanTajonar García, KarenHenning, JoergTakagi, YasuakiHenriques, JoaquimTakahashi, HideakiHensel, EnieTakahashi-Omoe, HiromiHeras-Molina, AnaTakeda, MasayukiHerbut, PiotrTakehiro, NishidaHerczeg, DávidTakeuchi, HirokiHerdt, Thomas H.Takizawa, ShuheiHernandez Medrano, JuanTamás Gergely, MolnárHernández, FuensantaTambella, Adolfo MariaHernandez, JesusTamburro, RobertoHernandez-Gomez, ObedTammen, ImkeHernández-Salazar, Laura TeresaTan, Tiong KaiHeroldová, MartaTan, ZhiliangHerrera Alsina, LeonelTang, XiaotianHerrera Haro, José GuadalupeTang, YulongHerrero, María JesúsTaniguchi, MasaakiHerring, AndyTao, FeifeiHerstad, Kristin Marie ValandTaoka, YousukeHerwig, EugeniaTaraba, Joseph L.Herzog, Catherine M.Tarlton, John FrancisHerzog, SvenTarricone, SimonaHess, ClaudiaTasker, LouisaHess, Tanja M.Tasoniero, GiuliaHibberd, Timothy J.Tassinari, PatriziaHiby, EllyTassone, SoniaHick, PaulTattoni, ClaraHickman, DebraTavares Pereira, MiguelHickson, RebeccaTaya, KazuyoshiHildebrandt, NicolaiTayari, HamasehHildreth, Michael B.Taylor, JimmyHill, FraserTaylor, MelanieHill, TomTedds, HelenHills, JamesTeixeira, AlfredoHiney, KrisTejeda, Juan F.Hinke, JeffersonTell, LisaHirsbrunner, GabrielaTellez-Isaias, GuillermoHirschenhauser, KatharinaTemple, DéborahHitachi, KeisukeTena, ElenaHittmair, Katharina M.Tenhagen, Bernd-AloisHockenhull, JoTerada, FuminoriHockenhull, JoannaTerler, GeorgHof, AnouschkaTerlouw, ClaudiaHof, Anouschka R.Terlouw, E. M. ClaudiaHofer, HeribertTernman, EmmaHoffman, MariaTerrado, JoseHogg, RachelTesi, MatteoHojo, TakuoTeske, ErikHołda, KarolinaThekkiniath, JoseHolland, Katrina E.Theuerkauf, JörnHolland, Kim N.Theurer, MilesHolland, MartinThibault, DelphineHollemans, Maarten S.Thibeaux, RomanHollinger, HannahThomas, DavidHolzhauer, MennoThompson, PeterHonda, MasahikoThompson, RileyHonda, MasatoThomson, DanHong, Cheng-YihThomson, JackHong, JinsuThomson, JenniferHood, JenniferThomson, Peter C.Hoog Antink, ChristophThorup, FlemmingHoppe, AndreasThulsiraj, VanessaHopper, Lydia MerielThymann, ThomasHopster-Iversen, CharlotteTian, QiyuHorák, PetrTiemann, IngaHoran, KateTiezzi, FrancescoHorback, KristinaTilbrook, AlanHornick, Jean-LucTimmons, Jennifer RumseyHorohov, DavidTinelli, AntonellaHorohov, David W.Ting, Ching-HuaHorowitz, AlexandraTinius, AlexanderHorst, Egon HenriqueTiplady, CatherineHorton, Brian J.Tizzani, PaoloHorvat, SimonTkachenko, HalynaHosseindoust, AbdolrezaTkacz, KatarzynaHotea, IonelaTlak Gajger, IvanaHou, FujiangTocci, RobertoHou, LinToepp, Angela J.Houde, PeterTofilski, AdamHoue, HansToftaker, IngridHoupt, Katherine AlbroTokarska, MałgorzataHoussier, MarianneTokuda, MasaharuHovinen, MariTokuyama, NahokoHowell, TiffaniTomassone, LauraHsieh, John C. F.Tomaszewska, EwaHsieh, KuangwenTombarkiewicz, BarbaraHsieh, Ming KunTomczuk, KrzysztofHu, JianyingTomczyk-Warunek, AgnieszkaHuang, ChangwenTong, JingouHuang, Jong-ChinToni, MattiaHuang, Wei-HsiangToomer, OndullaHuang, ZuyiToribio, RamiroHubbell, JohnToro Mujica, PaulaHuber, KorinnaTorre, Maria LuisaHuck, MarenTorres, Ana CatarinaHuertas, MarTorres, CristianHufschmid, JasminTorricelli, MartinaHughes, KateTóth, TamásHuijser, Marcel P.Touzot-Jourde, GwenolaHulsman Hanna, Lauren L.Toyota, KenjiHumm, Karen R.Tozaki, TeruakiHuntingford, JaniceTrabalza-Marinucci, MassimoHuntington, Gerald B.Trachsel, MaryHuo, Bing-XingTranfield, ErinHur, Tai-YoungTråvén, MadeleineHusnik, RomanTremolada, GiovanniHuson, Kathryn M.Trewavas, Anthony J.Hussein, MaythamTriay-Portella, RaülIacono, EleonoraTribe, AndrewIaffaldano, NicolaiaTroan, Brigid VictoriaIaria, CarmeloTroedsson, Mats H. T.Iarussi, FabrizioTrombetta, Maria FedericaIbáñez, Alberto HorcadaTroncarelli, Marcella ZampoliIbeagha-Awemu, Eveline MengwiTrotter, MarkIbrahim, MarwaTruu, JaakIburg, Tine MoesgaardTryjanowski, PiotrIchinohe, ToshiyoshiTsakmakidis, Ioannis A.Ijsseldijk, Lonneke L.Tsang, Susan M.Illek, JosefTschoner, TheresaIllera, Juan CarlosTsuji, YamatoImai, TadashiTsuka, TakeshiInfascelli, FedericoTsukahara, TakamitsuIngham, Aaron B.Tsukiyama-Kohara, KyokoInglis, G. DouglasTsuruta, ShogoInic-Kanada, AleksandraTucciarone, Claudia MariaIniesta-Cuerda, MaríaTudisco, RaffaellaIp, HonTufarelli, VincenzoIqbal, YasirTunick, MichaelIrvine, LeslieTuratto, MassimoIshikawa, AkiraTurcsán, BorbálaIshikawa, Marcia MayumiTurło, Agnieszka J.Islam, Md ZohorulTurner, Patricia V.Itami, TakaharuTurni, ConnyItani, KhaledTuśnio, AnnaItze-Mayrhofer, CorinaTuz, RyszardItzkowitz, MurrayTydén, EvaIussich, SelinaTylan, CatherineIvanković, AnteTyne, Julian A.Ivashchenko, AnatoliyTzika, EleniIvshina, IrenaUberoi, AayushiIwamaru, YoshifumiUccheddu, StefaniaIwata, HisatakaUdala, JanIzquierdo, MercedesUeckermann, EddieJabbar, AbdulUenoyama, YoshihisaJaber, José RaduanUlrich, ReinerJach, MonikaUnderwood, Keith R.Jack, Katharine M.Üney, KamilJackson, JohnUpton, SarahJalali, Beenu MozaUrios, VicenteJames, CharlotteUrrutia, OlaiaJamieson, La Toya J.Uwiera, RichardJamil, TariqUyeno, YutakaJanczak, Andrew M.Väärikkälä, SofiaJaneczek, MaciejVacher, Jean-PierreJang, Jae-CheolValaja, JarmoJang, Young DalValasi, IreneJaniszewski, PiotrValastro, CarmelaJankowiak, HannaValcácia Silva, SildivaneJankowski, JanValdés-Baizabal, CatalinaJanowicz, AnnaValente, TiagoJanowski, TomaszValentini, SimonaJansson, DésiréeValenzuela, CarolinaJanuś, EwaValenzuela-Galván, DavidJanus, IzabelaValergakis, GeorgiosJasińska, Karolina D.Valerio, ZupoJastezębska, EwaValero, YulemaJavǔrková, Veronika GvoždíkováValgaeren, BonnieJawor, PaulinaValiakos, GeorgeJaworska, JoannaValigurová, Andrea BardůnekJean Laven, LindaVallée, EmilieJelinski, MurrayValsecchi, Paola MariaJensen, KirstyValtolina, ChiaraJensen, Rasmus BovbjergValverde Piedra, Jose LuisJerez, NancyValverde, AlexanderJerry, CarmenVan De Vis, HansJesudhasan, PalmyVan Den Boom, RobinJesus, FatimaVan Der Aar, PietJewell, Dennis E.Van Der Staay, F. JosefJezierski, Tadeusz A.Van Der Vekens, ElkeJia, BeibeiVan Dixhoorn, IngridJiang, KangfengVan Eerdenburg, FrankJiménez-Peñuela, JéssicaVan Emon, MeganJiří, PatokaVan Erp-van Der Kooij, LennyJobling, MalcolmVan Kesteren, FreyaJohansson, Anna MariaVan Klink, Ed G. M.Johnson, Dylan M.Van Laar, HarmenJohnson, Kate F.Van Luijk, JudithJohnson, NicholasVan Marle-Köster, EsteJohnson, PaulVan Niekerk, TheaJonas, ElisabethVan Opzeeland, Ilse C.Jones, BrianVan Rensburg, C. JansenJones, KarinaVan Rongen, EricJones, Ronald S.Van Rooy, DianeJones-McVey, RosalieVan Santen, VickyJong, ChianjuVan Soom, AnnJoo, Hwang-SooVan Staaveren, NienkeJordan, BrianVan Steenbeek, Frank G.Jordan, GebhardtVan Wettere, WilliamJorge, ErikaVandenbussche, FrankJorgensen, MatthewVanderplaschen, AlainJorquera-Chavez, MariaVannier-Santos, Marcos AndreJoyce, PaulVannucchi, Camila InfantosiJu, Jyh-CherngVanselow, JensJu, Tz ChuenVapniarsky Arzi, NataliaJugo, Begoña M.Vardasca, RicardoJulien, DandrieuxVarejão, Artur SeveroJulliand, VéroniqueVarga, Zoltan M.Jung, LisaVargas-Bello-Pérez, EinarJung, Tae-SungVarga-Visi, EvaJuodka, RobertasVarner, Dana M.Juráček, MiroslavVarricchio, EttoreKaba, JaroslawVascellari, MartaKahilainen, Kimmo K.Vaughn, Mathew A.Kaji, KoichiVázquez Bringas, Francisco JoséKajihara, MasahiroVázquez-Domínguez, EvaristoKakehashi, RyosukeVázquez-Gómez, MartaKakinuma, MikiVeasey, JakeKalaitzakis, EmmanouilVecchiato, Carla GiudittaKalscheur, KennethVecchione, MichaelKamaldinov, Evgeniy VarisovichVečeřek, VladimírKamaszewski, MaciejVeerasamy, SejianKamiński, TadeuszVeiga Dos Santos, MarcosKanankege, Kaushi S. T.Veiga, InêsKaneko, GenVélez, Emilio J.Kanemaki, NobuyukiVéliz Deras, Francisco GerardoKang, JinheVenâncio, CátiaKania, BogdanVenancio, Emerson JoséKantor, MihailVenditti, MassimoKarakavuk, MuhammetVeneziani, MarioKaramon, JacekVenter, EstelleKarasova, Jana ZdarovaVentrella, DomenicoKaratzia, Maria-AnastasiaVentura, BethKarcher, DarrinVera Ávila, Héctor RaymundoKarenina, KarinaVera Rodríguez, ManelKareskoski, MariaVerardo, Lucas LimaKargar, ShahryarVéras, Antonia Sherlânea ChavesKaripidis, KenVerhoeven, MichaelKarpiński, MirosławVerissimo, CarolinaKarselis, GeorgeVerrinder, JoyKasapidou, EleniVerruck, SilvaniKasarda, RadovanVersaci, MarioKasimanickam, RamanathanVervaecke, HildeKasman, LauraViana, Joao Henrique MoreiraKasperek, KornelVicino, Greg A.Kasper-Völkl, ClaudiaViegas, CarlosKasprowicz-Potocka, MałgorzataVieira, CeferinaKasprzak, RobertVieira, Frederico Márcio CorrêaKasprzyk, AnnaVieira, Mônica LarucciKasprzykowski, ZbigniewVigo, DanieleKästner, SabineVigors, StaffordKaszak, IlonaVilar Ares, Maria J.Katsafadou, AngelikiVilhena, ManuelaKatsoulos, Panagiotis D.Villa, LucaKaukonen, EijaVillagómez, Daniel A. F.Kausar, TusneemVillanova, Fabiola ElizabethKavaliauskas, PovilasVillari, SaraKawasaki, KiyonoriVillarruel-López, AngélicaKawęcka, AldonaVillerd, JeanKędzierski, WitoldVincent, Antony T.Keegan, Jason D.Vincenti, LeilaKeele, John W.Vincenzetti, SilviaKeim, Juan PabloVinciguerra, Maria TeresaKeiper, Brett D.Vinogradov, SzergejKeisuke, KoyamaViñuela, JavierKelemen, Evan P.Violeta, RazmaitėKemper, NicoleViscardi, AbbieKemski, MeganViscasillas, JaimeKendall, NigelVisser, CarinaKentaro, YamadaVisser, KathalijneKeogh, KateVitale, AugustoKerem, DanVitale, Kristyn R.Kerlin, DouglasVitoria, ArantzaKerr, Caroline AudreyViuda-Martos, ManuelKesäniemi, Jenni EmiliaVizcaíno, Antonio JesúsKettunen, HanneleVizzarri, FrancescoKeuling, OliverVogt, Miriam AnnikaKhaliduzzaman, AlinVoigt, ChristianKhalil, Mahmoud MohamedVoigt, KatjaKhalil, Wael A.Volfova, MartinaKhan, Firdous A.Volsche, ShellyKholová, IvanaVon Eugen, KayaKhoo, ShaunVonk, JenniferKhurshid, ChrowVos, Peter L. A. M.Kiczorowska, BożenaVoslářová, EvaKidd, Jeffrey M.Vötterl, Julia C.Kidd, LisaVrba, JaroslavKiełbowicz, ZdzisławVukasinovic, NataschaKielland, KnutVullo, CeciliaKiełpińska, JolantaWaagbø, RuneKienapfel, KathrinWagener, KarenKiezun, MartaWagner, FranziskaKikusato, MotoiWagner, LianeKilada, Raouf W. S.Wahli, ThomasKiley-Worthington, MartheWähner, MartinKilroy, DavidWaine, KatieKim, Beob GyunWalker, Brian G.Kim, Hyeon TaeWalker, Julie AnnKim, InhoWalker, Terence I.Kim, Jong GugWall, HelenaKim, JunmoWaller, UweKim, KwangilWalsh, CarolynKim, Kyoung HoonWalter, Jasmine A.Kim, Min JungWalugembe, MuhammedKim, SuhkmannWamwayi, HenryKim, Sung-JoWan, Ho YiKim, SungwooWanapat, MethaKim, WooWandesforde-Smith, GeoffreyKim, Woo-HyunWang, ChangluKimminau, Emily A.Wang, Fun-InKind, Karen L.Wang, HongningKinder, James E.Wang, HuchengKing, AllanWang, LifeiKing, MeaganWang, QishanKing, MikeWang, Shu-YinKing, Sarah R. B.Wang, SongboKing, Stephanie EvelynWang, WeiminKino, TabitoWang, WenceKirczuk, LucynaWang, XiaoKirnan, JeanWang, YuxiKiros, Tadele G.Wang, ZhishengKirschvink, NathalieWard, Samantha J.Kirtiklis, LechWarner, DanielKishigami, SatoshiWarwick, CliffordKitazumi, AiWaszkiewicz, EwaKitchener, AndrewWaterhouse, TonyKittelsen, Käthe EliseWatkins, CraigKittl, SonjaWatson, AndreaKjærgaard, BenedictWazed, Md AbdulKjørsvik, ElinWeaver, AliceKjos, Nils PetterWebb, Edward C.Klammsteiner, ThomasWebb, Stephen C.Klaoudatos, DimitriosWeber, GregKlein, Joshua D.Webster, JohnKlein, WilfriedWeese, ScottKlich, DanielWeigel, Kent A.Kliver, SergeiWeigmann, SimonKlockiewicz, MaciejWeimer, Shawna L.Klos, MatthewWeiss, MartinaKluess, JeannetteWellington, MichaelKlunemann, MartinaWells, DeborahKmiecik, MichailWenk, CasparKnapczyk-Stwora, KatarzynaWerner, MarianneKnauer, WhitneyWerth, Alexander J.Knierim, UteWeston, Michael A.Knight, AndrewWestreicher-Kristen, EdwinKnight, JimWetscherek, WolfgangKnight, PeterWhang, KwangyounKnight, Walter E.Whitaker, JustineKnizkova, IvanaWhitehouse, NancyKnubben-Schweizer, GabrielaWhitehouse-Tedd, KatherineKobayashi, YasuhisaWhiting, TerryKobek-Kjeldager, CecilieWhittaker, AlexandraKoblmüller, StephanWichert, BrigittaKoch, ChristianWick, MacdonaldKoch, FranziskaWięcek, JustynaKochan, JoannaWiegard, MechthildKochanowski, MaciejWieland, MatthiasKoester, DianaWierzbicka, MałgorzataKogut, MichaelWierzbowska, IzabelaKohda, TakashiWigham, Eleanor E.Kohlmeier, PhilipWilk, IzabelaKöhne, MartinWille, MichelleKokokiris, LambrosWilliams, ByronKokoszyński, DariuszWilliams, Charlotte AmdiKokou, FotiniWilliams, David A.Kolakshyapati, ManishaWilliams, EllenKolenda, MagdalenaWilliams, Jane M.Kolisko, MartinWilliams, LeahKolora, RohitWilliams, ManodKoltes, DawnWilliams, SusanKomaki, ShoheiWills, AlisonKomisarek, JolantaWilson, Gary J.Komiyama, TomoyoshiWilson, GeorgeKomosa, MarcinWilson, MeganKondera, ElżbietaWiltschko, RoswithaKong, XiangfengWimbush, KarenKonieczka, PawełWinder, CharlotteKonold, TimmWindisch, WilhelmKonstantinos, MountzourisWiniarczyk, DagmaraKook, Peter HendrikWiniarska-Mieczan, AnnaKopecký, OldřichWinter, SilviaKopp, StevenWirth, WytammaKopper, Jamie J.Wischhusen, PaulineKordan, WładysławWisenden, Brian D.Kordowitzki, PawełWishart, JaneKorec, EvzenWiszniewska-Łaszczych, AgnieszkaKorine, CarmiWitarski, WojciechKornaś, SławomirWiteska, MałgorzataKorpysa-Dzirba, WeronikaWitkowska, DorotaKorzekwa, Anna J.Witkowska-Piłaszewicz, OlgaKosacka, JoannaWittek, ThomasKoseniuk, AnnaWlazło, ŁukaszKosik-Bogacka, DanutaWnuk-Pawlak, ElżbietaKosová, KláraWohlers, JeniferKostomitsopoulos, Nikolaos G.Wójcik, AnnaKot, KarolinaWojtacka, JoannaKotrschal, KurtWojtaś, JustynaKotsampasi, BasilikiWójtowski, JacekKotzamanis, YannisWojtusik, JessyeKougioumtzis, AlexandrosWolf, PetraKovitvadhi, AttawitWolfensohn, SarahKowalczuk-Vasilev, EdytaWolkers, Willem F.Kowalczyk, AlicjaWolny-Koładka, KatarzynaKowalczyk, MarekWong, Marty Kwok-ShingKowalczyk, PawełWong, Paul W. C.Kowalik, Magdalena K.Wood, JeffKrakowski, LeszekWoodard, Lauren E.Kralik, ZlataWoodburn, Maureen I. A.Kramer, Laura HelenWoodward, ElizabethKrasheninnikova, AnastasiaWoodward, HollyKrauel, Jennifer J.Woodworth, Jason C.Krauze, MagdalenaWoog, FriederikeKrauze-Gryz, DagnyWright, Tom C.Krebs, Bethany L.Wu, Chi-ReiKreft, StefanWutke, SaskiaKreisler, RachaelWydooghe, ElineKreuzer, MichaelWyffels, SamuelKrichbaum, SarahWyrostek, AnnaKrieg, JochenWysocki, MarianKrieger, MargretWysokińska, AnnaKrijger, Inge M.Xiao, MeitianKrishnamoorthy, ParamanandhamXie, QingmeiKristen, ErwinXie, ShangzheKristiansen, Tore S.Xu, JieKřížová, LudmilaXu, JingjingKrol, BarbaraXue, BaiKról, JolantaYadavalli, RaghavendraKronqvist, CeciliaYamaguchi, MasaruKrupa, EmilYamamoto, TakeshiKruszynski, WojciechYamamoto, TomofumiKrzysiak, Michał K.Yamasaki, JillKrzywonos, MałgorzataYamashita, KazutoKubacki, JakubYamazaki, TsuyoshiKubiatko, MilanYan, HuichaoKubota, KeiichiYan, QiKuchar, MartinYanagisawa, NaokoKucharczyk, DariuszYang, QienKucharska, KarolinaYang, RunjunKucheryavyy, AleksandrYang, Shyi-KuenKues, Wilfried A.Yang, Wei-HsiungKuhnke, SandraYang, WenzhuKulkarni, RaviYao, ChaoqunKulus, MagdalenaYasuno, NatsuruKumar, PraveenYe, LeiKumari, MeenaYeates, JamesKuo, Szu-ChengYerbury, RachelKupczyński, RobertYeste, MarcKurowicka, BeataYeung, PollyKuźnicka, EwaYıgın, Cahide Çiǧdem ErdemirKwiatek, KrzysztofYin, HsinbaiKwon, Jung-HoonYin, TongKwon, Woo-SungYngvesson, JennyLaarman, AnneYokoyama, SaichiroLabens, RaphaelYork, DanielLaciny, AliceYoshida, ShingoLackner, JuliaYou, XinxinLackner, ReinhardYoulatos, DionisiosLadewig, JanYousefi, MortezaLadine, Troy A.Yravedra, JoséLadwig-Wiegard, MechthildYu, HsiangLafferty, DianaYu, Il-JeoungLaffin, MichaelZąbek, KatarzynaLai, OlimpiaZąbek, TomaszLaliotis, George P.Zabielski, RomualdLaloë, Jacques-OlivierZachariah, Trevor T.Lam, StephanieZachut, MayaLamas-Toranzo, IsmaelZajac, MarzenaLambton, Sarah L.Żak-Bochenek, AgnieszkaLamichhane, PrabinŻakowska-Biemans, SylwiaLammi, Mikko J.Zaluski, RodrigoLandete-Castillejos, TomásZamaratskaia, GaliaLandi, NicolaZamora-Bustillos, RobertoLandim, Fernanda Da CruzZamora-Camacho, Francisco JavierLandim-Alvarenga, Fernanda Da CruzZampiga, MarcoLangley-Hobbs, Sorrel L.Zanetti, DeigoLanza, MassimilianoZanetti, Marcus AntonioLapierre, LisetteZani, DavideLappalainen, Anu K.Zannoni, AugustaLapuente, DennisZarantoniello, MatteoLara, Leonardo José CamargosZarazaga, Luis A.Lares, FernandoŻarczyńska, KatarzynaLari, EbrahimZardus, John D.Larivière-Gauthier, GuillaumeŻarski, DanielLarrondo, CristianZatoń-Dobrowolska, MagdalenaLarzul, CatherineZaworska-Zakrzewska, AnitaLasagna, EmilianoZdeněk, ŽertLathan, PattyZedler, PaulLatoch, AgnieszkaZeiler, GarethLau, QuintinZelli, RiccardoLaudadio, VitoZelmer, Derek A.Laurà, RosariaZemanova, MiriamLauriault, LeonardZenetos, ArgyroLavazza, AntonioZeng, Chang-JunLaw, BradleyZeng, QiufengLawson, CharlesZerani, MassimoLay, Donald C.Zhang, FanLázaro, Victoria LuñoZhang, GuanshiLazarowski, LuciaZhang, GuiguoLazzari, RafaelZhang, KeyingLe Gall, TonyZhang, LongxianLe Jeune, Sarah S.Zhang, MichaelLe Mieux, Frederick M.Zhang, ShihaiLe, Hieu HuuZhang, XiaoyuLecorps, BenjaminZhang, YongliangLedwoń, AleksandraZhang, ZheLee, Chang-KyuZhao, GuangyongLee, Jess G. H.Zhao, ShengguoLee, KichangZhou, GuangbinLee, KippeumZhou, HuitongLee, Kyung-WooZhou, JianweiLee, Sang-HeeZhou, QicunLee, Sang-SukZicarelli, LuigiLee, Su A.Zięba, GrzegorzLee, SungsillZięba-Przybylska, DorotaLee, Won YoungZiętek, JerzyLee, WonchoelZilic, ZeljkoLeeds, AustinZimmerman, Patrick H.Lees, Angela M.Zimmermann, PeterLegros, VincentZinn, StevenLei, Tze-HuanZinovieva, NataliaLeite, Fábio Pereira LeivasZiomek, MonikaLeliveld, Lisette M. C.Zitek, AndreasLeme, Ligiane De OliveiraZmijewska, AgataLemiere, StephaneZnamirowska, AgataLemke, NeyZoidis, EvangelosLeng, JoyŻółkiewski, PawełLeonardo, LeonardiZou, EnminLepczyński, AdamZou, JixingLepri, ElvioZsoldos, RebekaLessire, FrançoiseZsolnai, AttilaLetko, AnnaZucca, EnricaLetra Mateus, TeresaŻukowski, KacperLeveau, Lucas M.Żulewska, JustynaLevrino, Gustavo A. MariaZumbo, AlessandroLevrino, Gustavo MaríaZybailov, Boris L.Levy, Steven


